# Updates on Lymphovascular Invasion in Breast Cancer

**DOI:** 10.3390/biomedicines11030968

**Published:** 2023-03-21

**Authors:** Elisabetta Kuhn, Donatella Gambini, Luca Despini, Dario Asnaghi, Letterio Runza, Stefano Ferrero

**Affiliations:** 1 Department of Biomedical Surgical and Dental Sciences, University of Milan, 20122 Milan, Italy; 2 Pathology Unit, Foundation IRCCS Ca’ Granda Ospedale Maggiore Policlinico, 20122 Milan, Italy; 3 Department of Neurorehabilitation Sciences, Casa di Cura Igea, 20129 Milan, Italy; 4 Breast Surgery Unit, Foundation IRCCS Ca’ Granda Ospedale Maggiore Policlinico, 20122 Milan, Italy; 5 Radiotherapy Unit, ASST Grande Ospedale Metropolitano Niguarda, 20162 Milan, Italy

**Keywords:** lymphovascular invasion, breast cancer, LVI, breast carcinoma, prognosis, angioinvasion

## Abstract

Traditionally, lymphovascular invasion (LVI) has represented one of the foremost pathological features of malignancy and has been associated with a worse prognosis in different cancers, including breast carcinoma. According to the most updated reporting protocols, the assessment of LVI is required in the pathology report of breast cancer surgical specimens. Importantly, strict histological criteria should be followed for LVI assessment, which nevertheless is encumbered by inconsistency in interpretation among pathologists, leading to significant interobserver variability and scarce reproducibility. Current guidelines for breast cancer indicate biological factors as the main determinants of oncological and radiation therapy, together with TNM staging and age. In clinical practice, the widespread use of genomic assays as a decision-making tool for hormone receptor-positive, HER2-negative breast cancer and the subsequent availability of a reliable prognostic predictor have likely scaled back interest in LVI’s predictive value. However, in selected cases, the presence of LVI impacts adjuvant therapy. This review summarizes current knowledge on LVI in breast cancer with regard to definition, histopathological assessment, its biological understanding, clinicopathological association, and therapeutic implications.

## 1. Introduction

Together with local invasion, one of the main defining characteristics of cancer is its spread capability, resulting in metastases. Notably, metastatic disease is the leading cause of death in cancer patients [1]. Before cancer cells metastasize to a secondary site, they must first enter and spread throughout the vasculature. Hence, lymphovascular invasion (LVI) and, to a much lesser extent, perineural and neural invasion are one of the biologic prerequisites of systemic spread and metastases.

The College of American Pathologists (CAP) recommends assessing and reporting LVI in all Cancer Protocols, the “gold standard” in cancer reporting [2]. Moreover, there are site-specific differences in LVI reporting, in particular with regard to the distinction between lymphatic and blood vessel invasion and the size of invaded vessels. In breast cancer (BC), it is not necessary to distinguish blood capillaries from lymphatic channels, but specifying the involvement of dermal vessels is suggested due to its close association with inflammatory breast carcinoma [3,4]. According to the St. Gallen International Expert Consensus Conference guidelines, it is also advised to specify the presence of “extensive” LVI, but the morphological criteria for its consistent definition are still equivocal [5]. Regardless, the diagnosis of LVI is based on Rosen’s histopathological criteria [6]. LVI has been associated with local recurrence, distant metastases, and a worse prognosis for BC patients [1,7,8,9].

Currently, the therapy of BC patients is mainly dictated by well-established biological biomarkers, including estrogen receptors (ER), progesterone receptors (PR), HER2, and the Ki-67 proliferation index, together with TNM staging and age. However, the presence of LVI may impact the indication for adjuvant therapy [10]. Specifically, LVI affects radiotherapy indications in early BC (eBC) patients. Moreover, adjuvant chemotherapy selection may be implemented by the adjunct of LVI in the decision-making process.

In this review paper, we critically summarize the state of the art on the main aspects of LVI in BC, its histological characteristics and possible pitfalls, its biological understanding, clinicopathological associations, and therapeutic implications.

## 2. Histological Characteristics of Lymphovascular Invasion

For a long time, the presence of LVI has been identified on an optical microscope, described by pathologists, and recognized as a histopathological feature of malignancy and metastasis potential [11]. LVI, also known as lymphatic invasion, vascular invasion, or angioinvasion, is defined as the presence of tumor emboli within an endothelium-lined space (lymphatic vessels or blood capillaries) without underlying smooth muscle and elastic fibers. The majority of lymphatics do not contain erythrocytes. Nevertheless, distinguishing between lymphatic and blood vessel invasion is particularly hard and lacks a documented prognostic impact in BC [12]. In addition, Mohammed et al. elegantly showed that the vast majority (more than 97%) of tumor vascular emboli in lymph node-negative BC are indeed within lymphatic channels and often coexist with blood vessel invasion, further supporting the worthlessness of this discrimination [1]. Coherently, the CAP currently does not recommend such a distinction [4].

Specifically, the CAP advises reporting the occurrence of LVI in resection specimens since BC is known to spread through the vascular pathway, like many other malignancies [4]. Moreover, according to the AJCC cancer staging manual, LVI has no impact on the primary tumor extension (T category) [13]. Analogously, after neoadjuvant treatment, when the only residual tumor is LVI, it is assigned a ypT0 stage but a partial response (pPR).

The assessment of LVI is a time-consuming task with considerable interobserver variability and low reproducibility [14,15]. As a consequence, the reported frequency of LVI in BCs varies substantially among different studies, ranging from 8.8% to 69.5% [16,17]. Likely, differences in the histologic evaluation and study populations also contribute to this apparent discordance.

### 2.1. Histological Diagnosis

**Figure 1:**
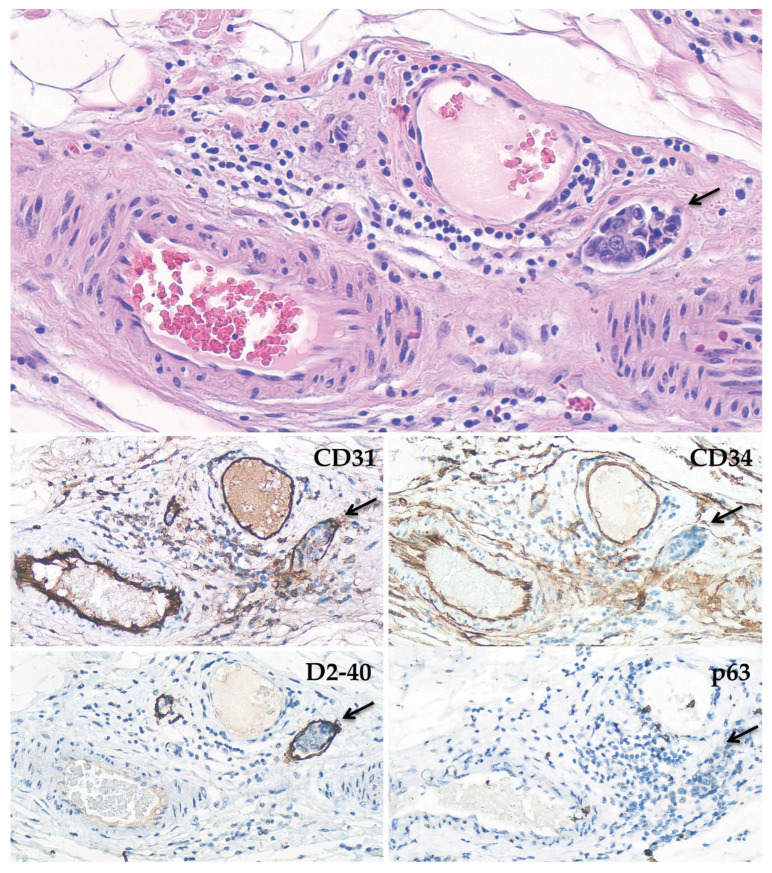
Representative pictures of an LVI tumor embolus (arrow) walled by an endothelial cell layer positive for CD31, CD34, and D2-40 immunostaining. p63 shows the absence of myoepithelial cells (200× magnification).

The assessment of LVI is routinely performed on hematoxylin–eosin-stained preparations, for which detailed histological criteria have been proposed and recommended for the diagnosis of LVI in breast pathology (Table 1 and Figure 1) [4,6].

LVI evaluation is more trustworthy in breast parenchyma outside, but close to, invasive carcinoma margins due to the difficulty in distinguishing true LVI from retraction artifact, mainly encountered within the tumor (see below) [6]. In addition, a few studies have shown that lymphangiogenesis is typically absent or reduced inside breast carcinoma, resulting in a significantly higher number of lymphatic vessels in the peritumoral stroma than in the intratumoral stroma [18,19,20]. Furthermore, Van den Eynden et al. found that only peritumoral LVI was significantly associated with node metastasis [17].

Histologically, the tumor emboli do not exactly shape the space they are in, leaving an intravascular empty space. Sometimes, this space is partially or completely occupied by fibrin clots and erythrocytes adhering to the tumor emboli, a consistent feature of the blood vessel LVI, or lymph content, composed of pinkish-stained homogenous material, more consistent with lymphatic channels [21]. In particular, the majority of lymphatics do not contain erythrocytes. Tumor emboli, on the other hand, can completely fill vascular spaces, mimicking in situ carcinoma [22].

Endothelial cells, which appear as a thin monolayer of cells with inconspicuous flat cytoplasm and homogenous elongated nuclei that can protrude in the vessel lumen, must line tumor emboli in LVI. Then, in breast parenchyma, usually small blood vessels (an arteriole and a venule), as well as a nerve trunk, are often found in close proximity to the LVI, although this finding is variable and inconsistent [6].

Cutaneous LVI is particularly relevant due to its strict association with the clinical entity of inflammatory breast carcinoma [3]. Histologic criteria for cutaneous LVI are the same as previously discussed. The main difference concerns the general lack of a small artery or vein close to the LVI. In addition, dermal LVI characteristically causes dilated lymphatic channels in the skin.

#### Lymphovascular Invasion Quantification

Although there is a lack of agreement about the precise criteria for classifying LVI, the St. Gallen International Expert Consensus Conference recommendations advocate quantifying LVI [2]. In order to determine the LVI extension, pathologists should report the number of foci per tissue block. Specifically, a seminal study by Colleoni et al. defined focal LVI as one focus in a single tumor block, moderate LVI as multiple foci in a single tumor block, and extensive LVI as one or more foci in multiple tumor blocks [8]. Relatedly, the CAP adopted a three-layer quantification method for LVI, including absent, present focal (LVI in one block only), and extensive (LVI in two or more blocks) [4].

### 2.2. Pitfalls

In the breast, LVI has been reported in combination with carcinoma but also with benign proliferative diseases. Specifically, one study by Eusebi and Azzopardi found non-neoplastic breast tubules within the artery or the venous wall in 5 of 50 consecutive cases of sclerosing adenosis [22]. In addition, fine needle aspiration or stereotactic techniques may result in the intravascular displacement of the benign epithelium [23,24,25,26], which may also be passively transferred to axillary lymph nodes following a biopsy or even breast massage [27,28]. However, in any case, these findings do not have a prognostic impact.

During specimen processing, tissue retraction creates artifactual clefts around nests of invasive and in situ carcinoma that can mimic LVI. Such retraction or shrinkage artifacts can be difficult to differentiate from true lymphovascular lumens [14]. Initially, the cause of the tissue retraction artifact has been referred to as “cold ischemia” due to delayed formalin fixation [29]. However, the following studies demonstrated the association of retraction clefts with tumor size, grade, LVI, peritumoral lymphatic vessel density, VEGFR-C expression, lymphatic tumor spread, and worse prognosis. For the authors, these findings corroborated the hypothesis that retraction clefts are not just a fixation artifact but the expression of an early stage of LVI [30,31]. Notably, the retraction artifact is more frequently observed in “no special type” carcinoma than in lobular carcinoma [30].

Exceptionally, breast carcinomas, as well as lymphomas, show a peculiar pattern of infiltration as the result of permeation of the pseudoangiomatous stromal hyperplasia (PASH), which simulates extensive LVI [32]. Immunohistochemistry can aid in the correct interpretation of similar cases.

### 2.3. Immunohistochemical Stainings

**Figure 2:**
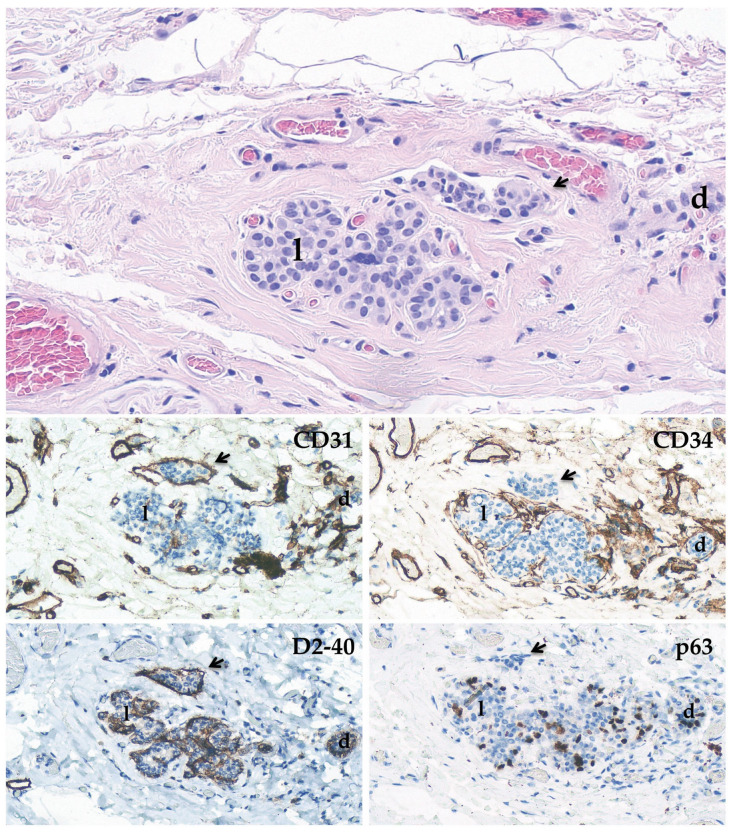
LVI (arrow) adjacent to a non-neoplastic lobule (l) and a small duct (d). The immunohistochemical staining demonstrates an endothelial layer (CD31+, CD34−, D2-40+, and p63−) surrounding the LVI embolus. Both the l and d show an irregular positivity for CD31, CD34, and faint D2-40 that can simulate LVI, particularly in small structures like d; however, positivity for p63 identifies myoepithelial cells of the terminal duct-lobular unit (200× magnification).

Immunohistochemical staining can aid in the identification and confirmation of LVI. The current wide availability of antibodies specific for antigens differentially expressed in small vessels allows for the identification of lymphatic channels, the distinction of blood capillaries, and the resolution of tricky morphologic dilemmas. Historically, many studies have described the utility of factor VIII, CD34, CD31, and Ulex europaeus agglutinin I in the identification of LVI endothelial cells [33]. However, factor VIII does not consistently mark endothelial cells, while CD31 and CD34 stain myofibroblasts, including those outlining nonvascular spaces as well as those typical of PASH [32]. In this setting, other endothelial immunostains were employed, such as D2-40, ERG, fli-1, and LYVE. Among these, D2-40, a podoplanin-specific antibody that highlights lymphatic endothelial cells in normal tissues, vascular neoplasias, and carcinoma-associated endothelial and epithelial cells, has demonstrated good utility and main diffusion [34,35]. As a result, a small panel of immunomarkers has been applied to distinguish lymphatics (D2-40+, CD31−/+, CD34−/+) from blood vessels (D2-40−, CD31+, CD34+) and their corresponding LVI [1]. However, Rabban et al. showed that D2-40 crossreacts with myoepithelial cells [35]. Aside from this, we observed that both normal and neoplastic ducts and lobules are sometimes circumferentially or partially surrounded by thin capillaries that, in the case of epithelial expansion, can simulate tumor embolism because the vessel lumen almost becomes virtual, giving a CD31+ or/and CD34+ linear stain instead of the circular, oval, or two-track stain appearance (Figure 2). Therefore, if a thin layer of cells is visible around a stamp cell cluster, it is always advisable to add at least one myoepithelial marker, such as p63 or others.

Again, the significant attenuation of the stretched endothelial cytoplasm in dilated lymphatic channels may be responsible for the apparent false-negative immunostaining of lymphatic endothelial cells [21]. Taking these findings together, caution should be taken while using immunohistochemical stains to identify LVI.

## 3. Biological Overview of Lymphovascular Invasion

LVI is a complex process composed of many steps. Initially, cancer cells must breach the epithelial basement membrane, invade the stroma, and reach the underlying vessel spaces [36]. These vessels can be preexisting or newly formed by tumor neoangiogenesis or neolymphangiogenesis [37]. Elegant in vitro co-culture systems demonstrated crosstalk between endothelial cells and BC cells with increased expression of angiogenic factors that maintain and stimulate the tumor vasculature. Therefore, endothelial cells have the ability to enhance the angiogenic potential of BC cells [38,39]. Moreover, tumor cells along the invasive front frequently display infiltrative activity, entering the surrounding tissue either as single cells, streaks, or clusters of cells.

Epithelial-to-mesenchymal transition (EMT), an evolutionarily conserved cellular program important for both physiological and pathological processes, including embryogenesis, wound healing, and tissue repair, plays a key role in carcinoma invasion [40]. Several microenvironmental stimuli promote tumor EMT by activating TGF-β and WNT signaling pathways, which in turn activate the EMT transcription factors of the Snail, Twist, and Zeb families [41]. These latter factors cause a substantial phenotypic switch in carcinoma cells triggered by cytoskeletal remodeling, increased expression of degradation protease, inhibiting cell-cell junctions, and cell polarity, which favor cell dissociation, mobility, and tumor invasion [41,42]. Indeed, EMT is a dynamic process where mesenchymal and epithelial features coexist and enable cancer cells to migrate in clusters rather than individually [3,41,43]. In fact, LVI typically appears as emboli or cell clusters on histologic examination.

An additional key player in cancer invasion is the extracellular matrix (ECM) [36]. In cancer, the ECM undergoes progressive remodeling by different cells, including cancer-associated fibroblasts (CAFs), cancer cells themselves, and tumor-associated macrophages (TAMs) [44,45,46]. In synthesis, specific ECM proteases, produced by both cancer and stromal cells, cleave TGF-β latent form to release its active form. Then, active TGF-β recruits and promotes CAF transformation from resident fibroblasts or other cells. CAFs contribute to both local tumor invasion and neoangiogenesis by producing growth factors and ECM proteins, in particular an oriented fibronectin matrix, which drives directional cancer cell migration [44,47,48]. Cancer cells may also produce and secrete several ECM components [45,46]. Similarly, TAMs synthesize and secrete TGF-β, proteases, and other signaling molecules that participate in the recruitment of CAFs as well as ECM degradation and remodeling. Moreover, TAMs adjacent to lymphatics promote neolymphangiogenesis and LVI, enhancing vessel hyperpermeability, dilatation, and disorganization [37,49].

Several lymphangiogenic factors are released by BC and stromal cells. Specifically, the most critical are VEGF-C and VEGF-D, which bind their receptor VEGFR-3 and stimulate lymphatic endothelial cells [50]. Both VEGF-C and VEGF-D undergo proteolytic maturation, which increases their affinity for VEGFR-3 and, consequently, lymphangiogenesis activity [51,52]. In addition, this VEGF-C maturation induces affinity for VEGFR-2 in the lymphatic and blood endothelial cells, contributing to lymphangiogenesis and possibly angiogenesis [53].

Once cancer cells reach the vessel wall, they must penetrate the perivascular cells, basement membrane, and endothelial cell layer. There is evidence that different mechanisms other than protease-mediated invasion contribute to cancer cell intravasation: (1) mechanical forces such as interstitial fluid pressure or contractile stress [54,55]; (2) CCR7-mediated chemotaxis of cancer cells toward endothelial cells [56,57]; (3) increased vessel permeability induced by cytokines or growth factors [58,59]; and (4) chemorepellent-induced defects (CCID) in endothelial cells [60]. Overall, LVI is the result of a finely tuned biological program that synergistically involves both cancer and stromal cells.

## 4. Clinicopathological Associations

Several studies have been conducted on neoplastic LVI in invasive breast carcinoma. Clinically, LVI has been consistently associated with younger patients [61,62,63,64,65,66]. Furthermore, isolated or rare studies found and reported an association between LVI and premenopausal state, symptomatic disease, BC-related lymphoedema, and a poor Nottingham prognostic index [12,67,68,69,70].

Then, LVI has been strongly associated with larger tumor size, higher histologic grade, and higher T staging [71,72]. Interestingly, “no special type” (previously called ductal) breast carcinoma has been shown to cause LVI more than other histotypes [1,12,72,73,74], while findings from two different studies had conflicting results regarding the association with tumor border type. Fisher et al. identified that LVI was significantly associated with tumors with stellate borders [73]; subsequently, the opposite finding, i.e., the association of LVI with pushing, nonstellate tumor borders, was reported by Davis et al. [75].

Based on increasing and consistent evidence, the presence of LVI indicates a worse prognosis for BC patients. LVI, in particular, has been shown to significantly predict the presence of lymph node metastases, including those in sentinel lymph nodes [71,72,74,76]. Moreover, LVI is a risk factor for local recurrence and distant metastases and a poor prognostic factor for both disease-free survival (DFS) and overall survival (OS) in BC patients. LVI seems to behave as a negative prognostic factor also in selected patient subgroups, such as patients with negative and positive lymph nodes analyzed separately.

Recent research has investigated the relationship between LVI and major predictive biological markers. LVI was found more frequently in tumors that were hormone receptor (HR)-negative, HER2-positive, and had a higher Ki-67 proliferation index [7,67,77]. According to the major intrinsic biological subtypes of BC, LVI is more common in HER2-enriched and luminal B-like subtypes [7,77].

### Lymphovascular Invasion in Heredo–Familial Breast Cancer

An interesting issue regards LVI in heredo–familial BC. Since young women have a higher incidence of LVI than older women [61,62,63,64,65,66], speculatively, a reasonable hypothesis to explicate such a finding could be a possible correlation with germline mutations, more expected in a younger cohort of patients than in older ones. Unfortunately, not many studies specifically analyzed this issue, and the available cumulative data came from a relatively small sample of BC patients.

Van Voss et al. analyzed and discussed the presence of LVI in 28 *BRCA1*-related BCs. Their expectation was the absence or scarcity of LVI in such cancers, mainly for supposed biological reasons [78]. First, *BRCA1*-related BCs are usually characterized by pushing borders, which they say is not a favorable condition for easily reaching vessels. However, this subjective statement is not supported by consistent scientific data and is disproved by one previous study that identified LVI as significantly associated with nonstellate tumors [75]. Second, *BRCA1*-related cancers are more often of a medullary-like histotype in comparison with sporadic BC, and such a histotype is usually characterized by little LVI. Despite these assumptions, the results showed LVI occurring in *BRCA1*-related BCs as often as in sporadic ones (25% versus 20.6%), leading to the conclusion that these BC cells could be able to overcome the apparent barrier to reaching vessels [78].

Furthermore, in a recent study, Atcı et al. showed a higher prevalence of LVI in *BRCA1* mutation carriers than in *BRCA2* and non-carriers (78% versus 54.1% and 55.3%, respectively) in a cohort of 302 patients, of which 75 were mutation carriers [79]. The authors discussed their results compared to the literature. Although some studies did not report a greater incidence of LVI in *BRCA1*- and *BRCA2*-related BCs, others showed instead a higher proportion, especially in *BRCA1* mutation carriers. Only one report described LVI as occurring more often in *BRCA2*-related than in *BRCA1*-related or sporadic BCs (53% versus 39% and 48%, respectively) [80].

The COPE study analyzed the characteristics of BC in *TP53* germline mutation carriers. LVI was described in comparison to other patient subgroups with early onset of BC [81]. LVI occurred in 33% of *TP53* mutation carriers, a higher percentage than *BRCA1* (14.5%), *BRCA2* (24.8%), and sporadic BC in young patients (19.9%). Curiously, LVI was found as being as common in *TP53* carrier BC as it was in HER2-amplified BC (34.6%). These results are not consistent with some previous findings, where much higher percentages of LVI were observed in *BRCA1*- and *BRCA2*-related BC subgroups [79].

## 5. Therapeutic Implications

Despite the fact that the negative prognostic value of LVI is well known, and that many studies have been conducted on its predictive value for better therapy choices, its inclusion in the decision-making process for BC standard care has not been adequately defined and is not acknowledged in most guidelines [82]. More recently, LVI was indicated for a better definition of radiotherapy protocols [83,84]. In particular, the St. Gallen International Consensus Guidelines recommend against partial breast irradiation in eBC patients with lobular carcinomas or when the LVI is present [83]. On the other hand, NCCN guidelines advise considering comprehensive regional nodal irradiation in patients with central/medial tumors, pT3 or pT2 tumors, and one of the following high-risk features: grade 3, LVI, or ER-negative [84].

In an adjuvant setting, a recent large French multicenter retrospective cohort study of over 17,000 patients found that LVI was an independent negative prognostic factor for DFS, OS and metastasis-free survival in all eBC patients, except both G3 and Luminal A-like BC treated with adjuvant CT. The authors concluded by considering a possible LVI positive predictive value for chemotherapy in certain subsets of eBC [7].

The introduction of genomic assays in clinical practice for HR-positive, HER2-negative BCs and the subsequent availability of a reliable prognostic tool could have scaled back interest in LVI-predictive value, making it less relevant, if not outdated, in the adjuvant clinical setting. Nevertheless, a new role for LVI as a parameter to employ in addition to genomic assays, especially when the scores are positioned in a gray zone for chemotherapy choice, has produced new updated studies. Regarding this, Makower et al. assessed the complementary role of LVI as an adjunct to a 21-gene recurrence score (RS) [85]. According to their findings, even though LVI correlated with a worse prognosis when RS ranged from 11 to 25, no chemotherapy benefit was observed in patients with LVI-positive BC compared to LVI-negative, indicating that LVI is not a useful parameter to add to 21-gene RS in such a setting [85].

Moreover, Mutai et al. analyzed a potential correlation between LVI and higher 21-gene RS [86]. Such a relationship was not identified, but they confirmed a negative prognostic value for LVI, especially in the group of patients with intermediate risk [86]. Similarly, Al-Zawi et al. have not found a statistically significant impact of LVI on 21-gene RS in a smaller sample of HR-positive and HER2-negative BCs [87].

Another related issue is the association between LVI and the presence of genes correlated with LVI among those included in the clinically available genomic assays. In this contest, Klahan et al. applied bioinformatics to investigate the mechanisms underlying LVI in BC patients, describing a very large number of genes differentially expressed between LVI-positive and LVI-negative BC patients, including 37 down-regulated and 49 up-regulated genes [88]. When such genes are compared to those included in the various genomic assays, only one correspondence is found (the *RPL37A* gene, which encodes for the ribosomal protein L37a, which is also present in the Endopredict^®^ assay). For the other assays, however, only some correspondence with genes from the same family was found (two genes for PAM50 and one for both OncotypeDx^®^ and Mammaprint^®^).

A field of more actual interest could be the LVI’s prognostic and predictive value in the neoadjuvant setting. The identification of LVI after neoadjuvant chemotherapy (NAC) seems to correlate with a worse outcome in many studies. Liu et al. described results from their retrospective analysis of 166 women treated with NAC [89]. In their multivariate analysis, LVI was identified in 45% of surgery samples and was significantly associated with decreased DFS and OS (HRs of 3.76 and 5.70, respectively, *p* < 0.01) [89]. When LVI was detected in triple-negative BC, it identified the subgroup with the poorest clinical outcome [89]. A concordant result has been subsequently published by Ryu et al. [90]. LVI was not only associated with worse OS (along with other factors such as mastectomy and HER2 overexpression), but it was also a significant independent negative prognostic factor in post-NAC patients, outperforming a pathological complete response. Once again, LVI in triple-negative BC seems to give the worst prognosis [90].

A larger cohort study of 1033 BCs confirmed the role of LVI in addition to other prognostic scores [91]. Particularly, study results confirmed that post-NAC LVI is an independent negative prognostic factor (for local recurrence, distant metastases, and OS), but also that LVI added accuracy to the other post-NAC prognostic scores considered. In their conclusions, the authors underlined the importance of LVI in the NAC setting, for example, in order to stratify patients eligible for subsequent adjuvant therapy. They also proposed the inclusion of LVI in the post-NAC scoring system [91].

## 6. Conclusions

LVI represents a major feature of biological aggressiveness among carcinomas. Coherently, strong evidence showed that LVI in BCs is associated with more aggressive clinicopathological characteristics, such as larger tumor size, higher histologic grade, higher T staging, and axillary lymph node metastasis. Importantly, LVI is an independent negative prognostic factor associated with local recurrence and distant metastasis as well as worse DFS and OS, even in lymph node-negative patients.

In clinical practice, the emergence of genomic assays as decision-making tools for adjuvant chemotherapy in early-stage HR-positive, HER2-negative BC patients have waned interest in LVI’s predictive power. However, sometimes genomic assays are not conclusive, and additional reliable prognostic parameters for chemotherapy selection could acquire relevance. In this situation, further studies on the potential use of LVI as an adjunct are wanted. Notably, genomic assays are expensive, burdening healthcare costs and causing socioeconomic disparities. Regardless, LVI is clinically important since it influences radiotherapy indications.

Given the strong prognostic power of LVI, standardized methods of LVI routine assessment with more reproducible outcomes are required to implement LVI use to better risk-stratify BC patients at no additional cost.

## Figures and Tables

**Table 1:** Criteria for the histologic diagnosis of lymphovascular invasion by Rosen et al. [6].

Criteria
LVI invasion must be located outside the margins of invasive carcinoma (extratumoral), usually peritumoral within 1 mm. LVI emboli typically do not perfectly shape the vascular space in which they are found, unlike invasive or in situ carcinoma. Endothelial cell nuclei should line the emboli space, otherwise, they likely represent a retraction artifact. Lymphatic channels often encircle or lie close to blood vessels.

## Data Availability

Not applicable.
